# Carbimazole induced acute generalised exanthematous pustulosis

**DOI:** 10.1186/2045-7022-5-S1-P16

**Published:** 2015-03-11

**Authors:** Madhavi Kadambi, santhosh K M, murali mohan BV, tousheed syed

**Affiliations:** 1 Narayana Health Mazumdar Shaw Multispecialty Hospital, bangalore; India; 2 Narayana Health Mazumdar Shaw Multispecialty Hospital, internal medicine, bangalore; India; 3 Narayana Health Mazumdar Shaw Multispecialty Hospital, respiratory medicine, Bangalore, India

## Background

Acute generalised exanthematous pustulosis (AGEP) also known as pustular drug eruption is an unusual drug induced hypersensitivity skin manifestation, characterized by acute onset of fever, and numerous non-follicular pin-head sterile pustules on erythematous background , usually over the flexural areas and face [1]. Neutrophils are involved in the pathogenesis of AGEP. Neutrophil-activating cytokines like IL-3 and IL-8 have been implicated [2]. Around 90 percent of the times AGEP is caused by a drug. Reported most often due to antibacterials. Carbimazole which is a thiourea antithyroid drug is not one of the usual suspect.

## Method

We report a case of a 50-year-old man with history of chronic diarrhea since 2 years, subsequently diagnosed as hyperthyroidism, without a previous history of psoriasis, developed a widespread, pin head pustular eruption on the face and extremities 48 hours after starting carbimazole for hyperthyroidism.His blood investigations revealed neutrophilic leucocytosis. The offending drug was stopped on day 3 and the pustules subsided by day 5. He was started on propylthiouracil instead of carbimazole, which he tolerated well. Patient refused skin biopsy and patch testing.

## Conclusion

Carbimazole in the past has been associated with ANCA associated vasculitis and vasculitic rash, urticaria, alopecia and maculopapular rashes. Carbimazole causing AGEP is not a common occurance. Only one case has been reported till now by Grange-Prunier et al in an 83 year old lady [2]. AGEP can often be confused with pustular psoriasis and clinical distinction between the two is difficult. Histologically the presence of spongiosis, eosinophilic exocytosis, apoptotic keratinocytes and minimal acanthosis may favor the diagnosis of AGEP [3], whilst the presence of acanthosis and papillomatosis may support pustular psoriasis.The most important finding is the disappearance of the rashes with discontinuation of the drug.

AGEP is a self limmiting disease with generalised sterile pustulosis. The clue to the diagnosis is the rapidity with which it developes after starting a drug. The short time for onset of lesions is thought to be due to previous sensitization. The course is usually uneventful and the patient usually recovers within few days of stopping the drug.

## Consent

Written informed consent was obtained from the patient for publication of this abstract and any accompanying images. A copy of the written consent is available for review by the Editor of this journal.
